# MYH7 R453C induced cardiac remodelling via activating TGF-β/Smad2/3, ERK1/2 and Nox4/ROS/NF-κB signalling pathways

**DOI:** 10.1098/rsob.230427

**Published:** 2024-06-12

**Authors:** Lingyu Wang, Linquan Li, Dazhong Zhao, Hongming Yuan, Huanyu Zhang, Jiahuan Chen, Daxin Pang, Yi Lu, Hongsheng Ouyang

**Affiliations:** ^1^ Key Lab for Zoonoses Research, Ministry of Education, College of Animal Sciences, Jilin University, Changchun 130062, People's Republic of China; ^2^ Chongqing Research Institute, Jilin University, Chongqing 401123, People's Republic of China; ^3^ Department of Human Genetics, Radboud University Medical Center, Nijmegen 6525 GA, The Netherlands

**Keywords:** hypertrophic cardiomyopathy, MYH7 R453C, cardiac remodelling, EGCG

## Abstract

Hypertrophic cardiomyopathy (HCM) is a monogenic cardiac disorder commonly induced by sarcomere gene mutations. However, the mechanism for HCM is not well defined. Here, we generated transgenic MYH7 R453C and MYH6 R453C piglets and found both developed typical cardiac hypertrophy. Unexpectedly, we found serious fibrosis and cardiomyocyte loss in the ventricular of MYH7 R453C, not MYH6 R453C piglets, similar to HCM patients. Then, RNA-seq analysis and western blotting identified the activation of ERK1/2 and PI3K-Akt pathways in MYH7 R453C. Moreover, we observed an increased expression of fetal genes and an excess of reactive oxygen species (ROS) in MYH7 R453C piglet models, which was produced by Nox4 and subsequently induced inflammatory response. Additionally, the phosphorylation levels of Smad2/3, ERK1/2 and NF-kB p65 proteins were elevated in cardiomyocytes with the MYH7 R453C mutation. Furthermore, epigallocatechin gallate, a natural bioactive compound, could be used as a drug to reduce cell death by adjusting significant downregulation of the protein expression of Bax and upregulated Bcl-2 levels in the H9C2 models with MYH7 R453C mutation. In conclusion, our study illustrated that TGF-β/Smad2/3, ERK1/2 and Nox4/ROS pathways have synergistic effects on cardiac remodelling and inflammation in MYH7 R453C mutation.

## 1. Introduction

Hypertrophic cardiomyopathy (HCM) is a hereditary cardiovascular disease affecting 1 in 500 people, while the penetrance of clinical HCM manifestations is heterogeneous, from asymptomatic to malignant remodelling [1]. HCM is the most usual cause of sudden death (SCD) in young people and athletes. The pathogenesis of HCM is complex and half of HCM patients bear one or more sarcomeric gene mutations [2]. MYH7 encodes the β-myosin heavy chain (β-MHC), the main myosin subtype of human cardiac ventricles, and it comprises around one-third of the sarcomere proteins [3]. HCM patients with the MYH7 mutations account for 20–40% of total clinical patients [2]. MYH6 encodes the alpha cardiac myosin heavy chain (α-MHC), which shares over 90% sequence homology with β-MHC [4].

The Arg453 site selected in this study is located within the highly conserved surface loop of the myosin motor domain within β-MHC, which lies between the nucleotide-binding pocket and the actin-binding site, connecting to other essential structural elements. During myocardial contraction, myosin heavy chain (α-MHC) is involved in the regulation and execution of muscle contraction by binding with actin [5]. The transition of Arg453 to Cys453 (R453C) affected the signal between actin and ATP binding sites, reduced ATPase activity and *in vitro* motility coupled with increased force production, as well as a 35% decrease in ATP binding rate and a threefold slowdown in the ATP hydrolysis step/recovery stroke, potentially implicating this step as the rate-limiting factor in the ATPase cycle [5,6]. Though the structure of human α-MHC remains undetermined, most hypotheses regarding the impact of MYH6 mutations on α-MHC structure are based on comparisons with the established structure of β-MHC [7]. Mice have been widely used as animal models for MYH7 mutations, even though α-MHC, rather than β-MHC, is the mainly expressed myosin protein in the ventricles [8]. Previous reports on homozygous mouse models with the R453C alpha-isoform (α-MHC) mutation presented an ~80% increase in the maximum ATPase, with a ~30% decrease in human β-cardiac subfragment 1 [9], highlighting the functional differences between isoforms and emphasizing the need for a larger mammalian animal model. The beta-isoform (β-MHC) is the main form in human ventricles, as is the case for swine [10]. In addition, studies on swine have recapitulated human calcium handling and revealed crucial signalling pathways for disease treatment [11]. Thus, swine can serve as an ideal animal model for simulating pathological manifestations and the study of HCM.

Cardiac fibrosis is a common feature among advanced cardiovascular diseases, and it greatly increases the occurrence of heart failure (HF) and CSD [12]. During the process, transforming growth factor β (TGF-β) isoforms play a critical mission in collagen synthesis. The canonical SMAD-dependent pathway occurs when TGF-β binds to TGF-β receptors to promote the transformation of cardiac fibroblasts to activated myofibroblasts [13]. Non‐canonical TGF‐β pathways involved activation of mitogen‐activated protein kinases (MAPKs), PI3K/AKT and NF-κB signalling pathway. The MAPK-extracellular signal-regulated kinase (ERK) signalling pathway and NF-κB signalling pathway have been reported to be involved in inflammation and oxidative stress. The activation of PI3K/AKT has been reported to promote cardiac fibrosis through augmenting cardiomyocyte apoptosis [14].

Oxidative stress is involved in the development of heart disease, such as cardiac remodelling, dysfunction and even HF. The accumulation of reactive oxygen species (ROS) has been proven to initiate endothelial damage, thus aggravating inflammation [15]. NADPH oxidases (NOXs) are specialized ROS-producing enzymes, and they are involved in angiogenesis, inflammation, hypertrophy and myocardial fibrosis [16]. In the heart, the overexpressed ROS is mainly regulated by the Nox2 and Nox4 isoforms; the activation of Nox2 needs extracellular cytokines, while Nox4 is constitutively activated and is regulated by its expression level [17].

HCM is a monogenic cardiac disorder commonly induced by sarcomere gene mutations. However, the mechanism for HCM is not well defined. Therefore, we generated MYH7/MYH6 R453C heterozygous piglet models and H9C2 MYH7 R453C mutation cell line with Anc-BE4 max and the same one gRNA concurrently targeting MYH7 and MYH6. By generating pig deficiency for MYH7/MYH6 gene, we demonstrated that loss of MYH7 (but not MYH6) results in serious fibrosis and cardiomyocyte loss at the animal level. Furthermore, the phosphorylated Smad2/3, ERK1/2 and NF-kB p65 proteins in the MYH7 R453C mutation cardiomyocyte are increased. These data provided direct evidence that MYH7 R453C induces cardiac remodelling via activating TGF-β/Smad2/3, ERK1/2 and Nox4/ROS/NF-κB signalling pathways.

## 2. Material and methods

### 2.1. Transfection of porcine fetal fibroblast cells

Resuscitated porcine fetal fibroblast cells (PFFs) were stored in our laboratory and cultured in DMEM (Gibco, Grand Island, NY, USA) with 10% fetal bovine serum. When the six-well plates reached 90% confuence, the cells were collected and electroporated with 25 μg of the AncBE4max vector and 20 μg sgRNA expression plasmid using an electroporation instrument, BTX ECM 2001 (Harvard Bioscience, Holliston, MA, USA) in 300 μl Opti-MEM (Gibco, Grand Island, NY, USA). The parameters were as follows: 3 pulses, 1 ms and 340 V for 1 repeat in 2 mm gap cuvettes. The electroporated PFFs were digested for genotyping after culturing at 37°C, 5% CO_2_ for 72 h.

### 2.2. Plasmid acquisition and vector construction

The AncBE4max and the pBluescriptSKII+U6-sgRNA(F+E) empty plasmids were purchased from Addgene (#112094, #74707). The empty pBluescriptSKII+U6-sgRNA (F+E) was chosen for its U6 promoter with BbsI sites driving sgRNA for insertion of a spacer sequence. Target sgRNAs were designed according to the genome from NCBI and synthesized by GENEWIZ. To construct an integrated sgRNA-expressing plasmid, single-stranded DNA was annealed to double-stranded DNA and then ligated to the notched U6-sgRNA vector, which was digested with BbsI overnight.

### 2.3. Somatic cell nuclear transfer

The protocol followed our previous publications [18]. In short, the first polar body was removed from mature oocytes, and then the donor cells were fused with enucleated oocytes by BTX electrofusion equipment. After approximately 16 h of culture, the reconstructed embryos were then transferred into synchronized recipient swine. Pregnancy was generally monitored 35 days later by ultrasonography. Eventually, there were six piglets with MYH7 R453C mutation, four piglets with MYH6 R453C and three wild-type (WT) piglets born. Two of the WT piglets were female, one was male. One of the piglets with genotype MYH6 R453C was female, three were male. Three of the piglets with genotype MYH7 R453C were female and three were male. Transgenic piglets were weaker than normal WT piglets after birth, were significantly lower in weight than WT piglets after birth and had respiratory murmurs. Two piglets with MYH7 R453C mutation and one with MYH6 R453C mutation died around 4 days after birth. The histopathology illustrates the hypertrophy remodelling within dead MYH7 R453C and dead MYH6 R453C, typically hypertrophic left ventricle and interventricular septal. By Sirius red staining, the hearts of the three dead piglets presented apparent fibrosis (electronic supplementary material, figure S1). The remaining piglets were euthanized on the fourth day after birth.

### 2.4. Histology and histochemical analyses

Hearts from swine were washed with PBS, and then were fixed in 4% paraformaldehyde at 4°C overnight. Fixed hearts were washed using PBS and were then paraffin-embedded. The tissue was cut into 5 µm sections for Masson dyeing, Sirius red staining and haematoxylin and eosin (HE staining) to measure the degree of cardiac fibrosis. Custom ImageJ software was used for image analysis.

### 2.5. Serologic detection

Blood samples from piglets were placed at 4° for 30 min, and the serum was prepared by centrifugation at 4℃ and 1500 *g* for 15 min. Finally, the supernatant was transferred to a 200 µl centrifuge tube, and all the plasma and serum samples were stored in a −80℃ refrigerator for further experimental study. We used ELISA kits from Nanjing Jiancheng to detect biochemical markers of myocardial injury, including serum creatine kinase (CK) isoenzymes and cardiac troponin (cTnT).

### 2.6. Transcriptome sequencing (RNA-Seq)

The ventricular tissue was preserved in liquid nitrogen, and RNA sequencing was performed and analysed by GENEWIZ Biotechnology Co. (Suzhou, China). Image analysis was conducted on the HiSeq instrument by use of the HiSeq Control Software (HCS) + OLB + GAPipeline-1.6 (Illumina).

### 2.7. Cell culture

H9C2 cells at passage 10 were purchased from the American Type Culture Collection (Manassas, VA, USA) and were cultured in DMEM medium with supplementation of 100 U/ml penicillin–streptomycin and 10% fetal bovine serum, and then incubated at 37°C, 5% CO_2_. When the cell growth density reached about 80–90%, they were subcultured for the next generation by 0.25% pancreatic enzyme.

### 2.8. Cell viability assay

Cell viability was determined using an MTT assay as described previously [19,20]. Ten microlitres of MTT solution (5 mg/ml) was introduced to the cells, followed by a further incubation period of 4 h. Subsequently, 100 µl of a formazan solubilizing solution was added, and the samples were incubated for an additional 4 h. The absorbance was then measured at a wavelength of 570 nm using a microplate reader (BioTek Instruments, Winooski, VT, USA).

### 2.9. Quantitative real-time polymerase chain reaction

Total RNA was extracted from the left ventricle of swine with TRIzol-A+ reagent (Tiangen), and cDNA was obtained with a kit (High-Capacity cDNA Reverse Transcription Kit, Tiangen). Three replicates were set for each group of PCR samples, and the procedure was as follows: 94°C, 15 s; 60°C, 30 s; and 72°C, 30 s at 40 cycles with the iQ5 Multicolor RT-PCR System. Primer sequences for GAPDH, TGF-β, β-MHC, type I collagen (Col1a1), natriuretic peptide A (NPPA), natriuretic peptide B (NPPB), collagen type III alpha 1 chain (Col3a1) and connective tissue growth factor (CTGF) are listed in electronic supplementary material, tables S1 and S2.

### 2.10. Western blotting

**Figure 1.**
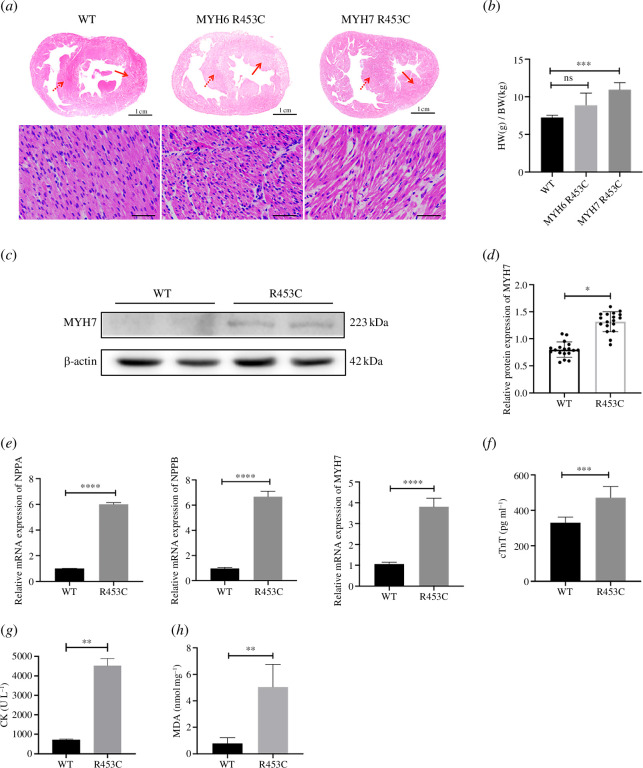
The MYH7 R453C (but not the MYH6 R453C) piglets developed premature myocardial injury and cardiac hypertrophy. (*a*) Representative picture of whole heart cross and local histological lesion of the left ventricle for litter WT, MYH6 R453C (M6) and MYH7 R453C (M7) piglets. The dashed red arrows indicate interventricular septal hypertrophy and the solid red arrows indicate left ventricle hypertrophy. (*b*) HW/BW of litter WT (*n* = 3), MYH6 R453C (*n* = 3) and MYH7 R453C (*n* = 4) piglets at post-natal 4 days. (*c*) Western blotting for MYH7 collected from the left ventricle of piglets with WT and MYH7 R453C mutation. (*d*) MYH7 expression quantified by densitometry and normalized to β-actin levels. (*e*) Relative mRNA expression of hypertrophy markers, NPPA, NPPB and MYH7 within the left ventricle of WT and MYH7 R453C piglets. (*f*) Serum cTnT level (ng/ml). (*g*) CK activity (U/l). (*h*) MDA level (nmol/mg).*p < 0.05, **p < 0.01, ***p < 0.001, ****p < 0.0001. *n* = 3 biologically independent samples.

**Figure 2.**
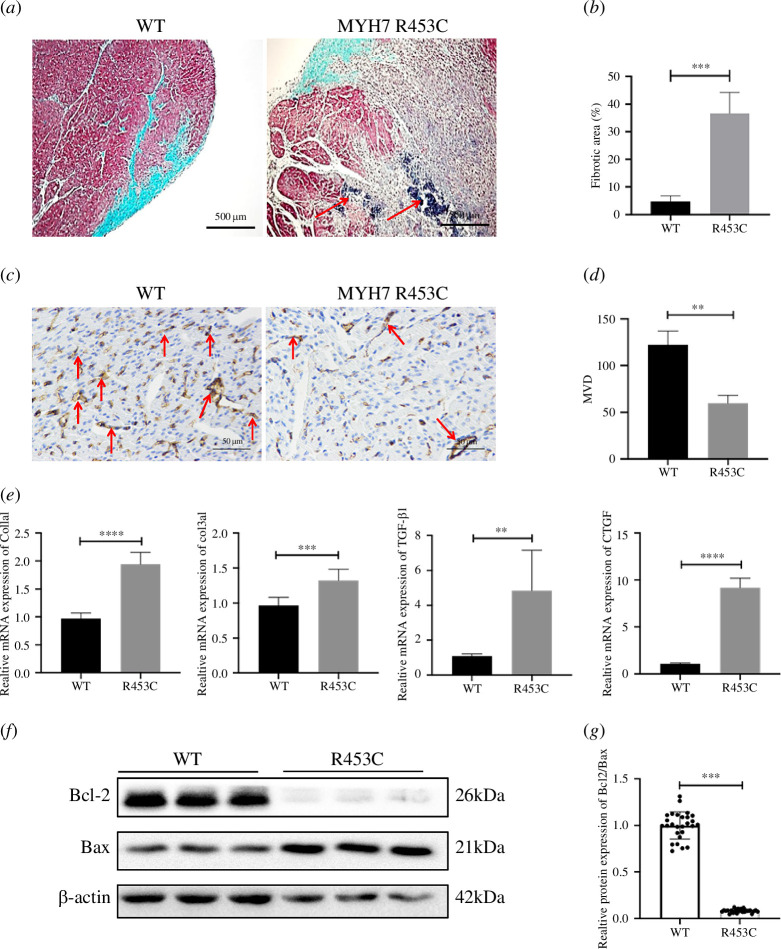
The MYH7 R453C developed malignant fibrotic remodelling. (*a*) Masson dyeing for the left ventricular of WT (*n* = 3) and MYH7 R453C (*n* = 4) piglets. Red arrows mean fibrosis and necrosis. The largely unstained region in the MYH7 R453C image means new collagen fibre. (*b*) Percentage of fibrotic area depicted in (*a*). (*c*, *d*) Immunohistochemical staining of the WT and MYH7 R453C piglets with CD31+ antibodies and their capillary density. The solid red arrows indicate microvessels. (*e*) Relative mRNA expression of Col1a1, Col3a1, TGF-β1 and CTGF. (*f*, *g*) The protein expression of Bcl-2 and Bax in left ventricle was detected by western blotting and their grey analysis value. **p < 0.01, ***p < 0.001, ****p < 0.0001. *n* = 3 biologically independent samples.

**Figure 3.**
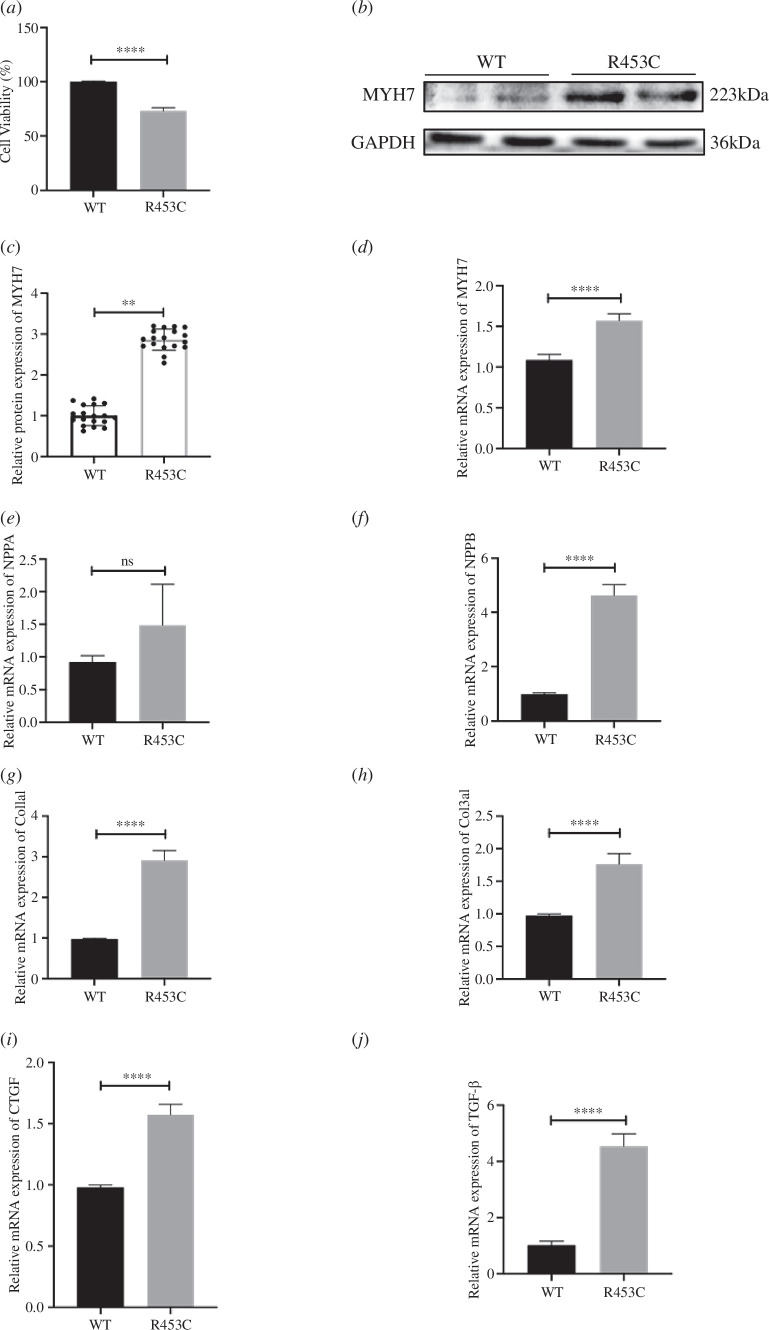
Effect of MYH7 R453C mutation on cell survival and expression levels of fetal gene program in H9C2 cells. (*a*) Effects of MYH7 R453C mutation on the H9C2 cell survival rate. (*b*, *c*) The protein expression of MYH7 within H9C2 with WT and MYH7 R453C. (*d*–*f*) Relative mRNA expression of hypertrophy markers, including NPPA, NPPB and MYH7. (*g*–*j*) Relative mRNA expression of fibrotic gene, Col1a1, Col3a1, TGF-β1 and CTGF. **p < 0.01, ****p < 0.0001. *n* = 3 biologically independent samples.

**Figure 4.**
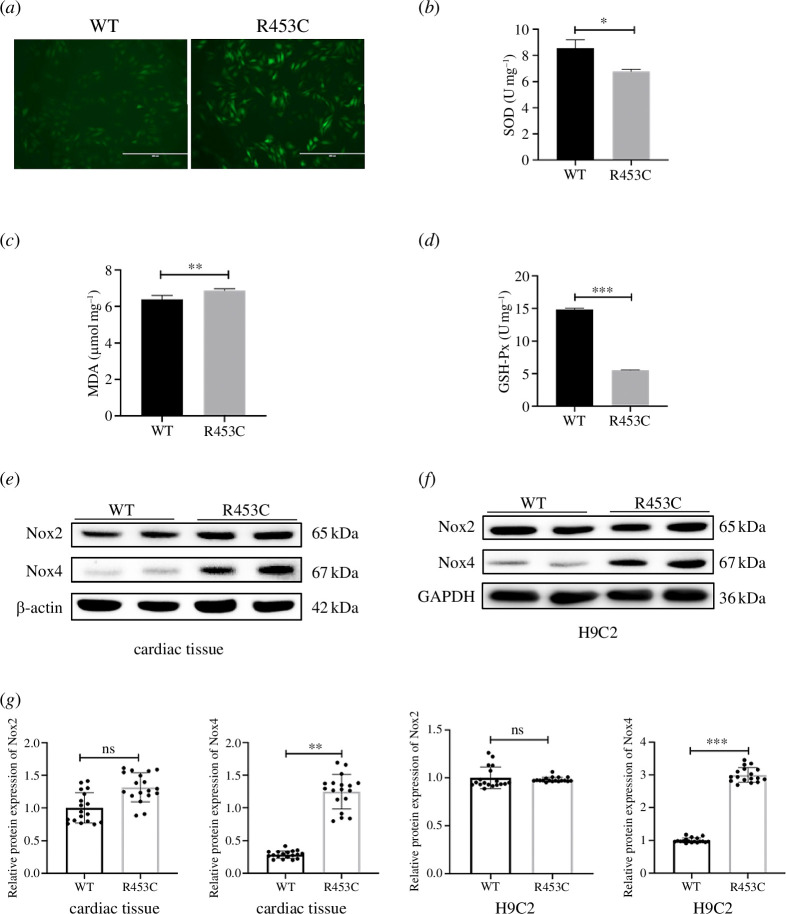
Effect of MYH7 R453C mutation on oxidative stress in cardiomyocytes. (*a*) Effect of MYH7 R453C mutation on ROS production (scale bar = 200 µm). (*b*–*d*) The expression level of SOD (*b*), MDA (*c*) and GSH-Px (*d*) in the MYH7 R453C mutant and WT H9C2 cell lines. (*e*, *f*). The protein expression of Nox2 and Nox4 in piglets' left ventricle (*e*) and H9C2 cell lines (*f*) are detected by western blotting. (*g*) Quantitative evaluation of Nox2 and Nox4 protein levels in piglets’ left ventricle and H9C2 cell lines. *p < 0.05, **p < 0.01, ***p < 0.001. *n* = 3 biologically independent samples.

**Figure 5.**
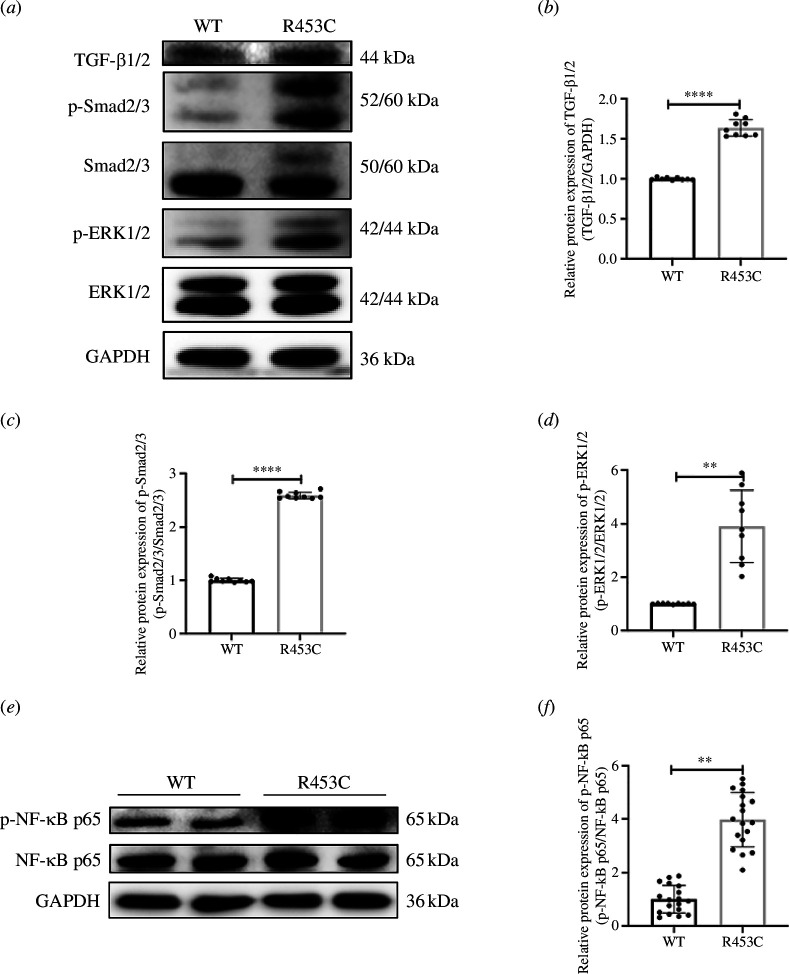
Effect of MYH7 R453C mutation on TGF-β/Smad2/3, ERK1/2, NF-κB and PI3K/AKT pathways. (*a*) The protein expression of TGF-β/Smad2/3 and ERK1/2 cascades were detected by western blotting. (*b*–*d*) The protein expression of TGF-β1/2, p-Smad2/3/Smad2/3 and p-ERK1/2/ERK1/2 were quantitated using densitometry. (*e*) The protein expression of NF-κB signalling was detected by western blotting. (*f*) Quantitative analysis of the protein expression of p-NF-κB p65 and NF-κB p65. **p < 0.01, ****p < 0.0001. *n* = 3 biologically independent samples.

The fresh samples from the left ventricle were resolved in lysis buffer and quantified by BCA kit (Beyotime, China). Using the SDS-PAGE Kit (Beyotime, China), protein samples were separated. Then, they were incubated with a primary antibody against TGF-β 1/2 Rabbit Polyclonal Antibody (#AF0297, Beyotime Biotechnology), ERK1/2 (#BM4326, Boster Biological Technology), p-ERK1/2 (#BM4156, Boster Biological Technology), SMAD2/SMAD3 Rabbit Polyclonal Antibody (#AF8001, Beyotime Biotechnology), Phospho-Smad2 (Ser465/467)+Smad3 (Ser423/425) Rabbit Polyclonal Antibody (#AF5920, Beyotime Biotechnology), Phospho-NF-κB p65 (Ser276) Rabbit Polyclonal Antibody (#AF5875, Beyotime Biotechnology), NF-kB p65 Rabbit Monoclonal Antibody (#AF1234, Beyotime Biotechnology), Anti-GAPDH Antibody (#A00227-1, Boster Biological Technology) and horseradish peroxidase-labelled secondary antibody afterwards (#abs20002, Absin Bioscience Inc. and #A0216, Boster Biological Technology). The bands in each group of western blotting images in figures 1*c*, 2*f*, 3*b*, 4*e,f* and *5e* are duplicates of the same sample. Three replicates of each western blotting experiment were performed. *n* = 3 biologically independent samples. All uncropped gels for western blotting are shown in electronic supplementary material, figure S2.

### 2.11. Detection of oxidative stress detection

The cells were seeded in a six-well plate with three duplicate wells in each group. DCFH-DA probe was diluted at 1:1000 with DMEM without phenol red. After the cells were attached to the wall, the culture medium was removed and replaced with the culture medium containing DCFH-DA probe, and they were incubated for 20–30 min at 37°C, 5% CO_2_. The excess DCFH-DA was removed by PBS 2–3 times. The intensity of fluorescence was detected and photographed, and the ROS fluorescence intensity was analysed by Image J. Malondialdehyde (MDA, Beyotime Biotechnology), superoxide dismutase (SOD, Beyotime Biotechnology) and glutathione peroxidase (GSH-Px, Beyotime Biotechnology) were measured according to the instructions.

### 2.12. Mutation detection and off-target detection

Mutation detection was performed as previously described [21]. The PCR primers for the sgRNA target sites were as follows: forward, 5′-GCCGACAAATCTGCCTACCT-3′ and reverse, 5′-CGAGGCAGCACCTTCTCAAT-3′ (fragment size of MYH6), forward, 5′-TGAAGCAGCGAGAAGAGC-3′ and reverse, 5′-GCAGGTCCATGCCAAAGT-3′ (fragment size of MYH7).

Using the website http://www.rgenome.net/cas-offinder/, we speculate about possible off-target sites, and the default condition is that the DNA mismatch number is ≤3, the DNA and RNA Bulge Size is 0.

### 2.13. Epigallocatechin gallate

Epigallocatechin gallate (EGCG) was purchased from MedChem Express (MCE, USA, HY-13653) with a purity of 99.87%. EGCG powder was dissolved in distilled water to form 40 mM EGCG storage solution, which was filtered by 0.22 μm aseptic filter membrane and then packaged and frozen in an ultra-low-temperature refrigerator for subsequent experiments.

### 2.14. Statistics analysis

Data are expressed as mean ± s.d., with at least three individual determinations in all experiments. Data of two group comparisons were determined by unpaired Student’s *t*‐test and multiple group comparisons were analysed with one-way ANOVA with Bonferroni’s post-tests using GraphPad Prism 8.0. *p*-values ≤0.05 represented statistical significance. All experiments were performed at least three times.

## 3. Results

### 3.1. Generation of MYH6/MYH7 R453C swine model

**Figure 6.**
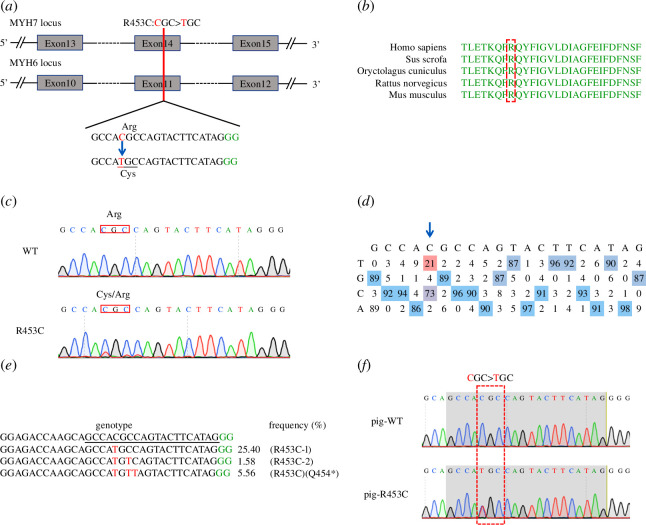
Generation of MYH7/MYH6 R453C mutant porcine. (*a*) The gRNA sequences targeting the MYH6 and MYH7 locus. The PAM sequence is green; gRNA-target sequences are black; substituted bases are red. (*b*) The MYH7/MYH6 R453C is highly conserved across human, swine, rabbit and rat. The targeted amino acid is framed in red. (*c*) Representative targeted sequencing chromatograms of WT and edited PFFs. The targeted and substituted nucleotides are circled in red. (*d*) The editing efficiency of the gRNA across the sequence on the target locus. The targeted nucleotide is under blue arrows. (*e*) The genotype and proportion of identified monoclonal cells, the substituted nucleotide is red; the PAM sequence is green. (*f*) PCR amplicons of the target site from genomic DNA of mutant piglets and sequenced.

Here, we designed a guide RNA (gRNA) targeted on c. 9123C>T MYH7/ MYH6 (figure 6*a*), which is highly conserved among species (figure 6*b*), and the MYH7/MYH6 R453C mutation is associated with malignant pathophysiology in patients with familial HCM. Upon delivering the optimized cytosine base editor Anc-BE4max and the gRNA into porcine fetal fibroblasts, we obtained a population of cells containing a single substitution of Arg to Cys on target (figure 6*c*), and the corresponding editing efficiency was around 21% (figure 6*d*). Finally, we obtained around 25.4% monoclonal cells with targeted c. 9123C>T MYH7/MYH6 mutation. The c.9123C>T and c.9125C>T MYH7/MYH6 mutation also encodes MYH6/MYH7 R453C, although the ratio of this genotype was only 1.58% (figure 6*e*). Finally, PFFs with c.9123C>T MYH7/MYH6 heterozygous mutations were chosen as the donor cells and subsequently underwent somatic nuclear transfer (SCNT). Genotypes and possible off-target sites of piglets were identified at birth (figure 6*f*; electronic supplementary material, table S3). Furthermore, no off-target mutations were detected at the five potential off-target sites in the MYH6/MYH7 R453C piglets (electronic supplementary material, figure S3). These results demonstrated that the cytosine base editor Anc-BE4max system is efficient for targeting the MYH7/MYH6 R453C in swine.

### 3.2. MYH7/MYH6 R453C piglets developed cardiac hypertrophy

As shown in figure 1*a*, the histopathology illustrates the hypertrophy remodelling within MYH7 R453C and MYH6 R453C, typically hypertrophic left ventricle and interventricular septal. The quantitative image analysis of hypertrophic left ventricle and interventricular septal is shown in electronic supplementary material, figure S4. HE staining showed that disarray of myofibres and myofibrillar loss could be clearly observed in piglets with MYH7 R453C, while the MYH6 R453C mutant presented no significant change. In addition, the raising of heart weight/body weight (HW/BW) further provides evidence for myocardial hypertrophy within the MYH7 R453C piglets (figure 1*b*). The specific piglets values of HW and BW are listed in electronic supplementary material, table S4. These results demonstrate that MYH7 R453C, not the MYH6 R453C induced myocardial injury and cardiac hypertrophy at an early stage. Thus, further analyses were performed on piglets with MYH7 R453C. The protein expression of beta-myosin heavy chain in piglets with MYH7 R453C mutation was significantly upregulated compared to WT while there was no significant increase in the WT piglets (figure 1*c,d*). To further verify the formation of premature hypertrophy, we evaluated the expression level of hypertrophy markers and found greatly increased mRNA expression of fetal genes *NPPA*, *NPPB* and *MYH7* within MYH7 R453C piglets (figure 1*e*). Furthermore, the MYH7 R453C piglets presented an abnormal increase in serum cTnT, CK and MDA compared with WT piglets (figure 1*f–h*).

### 3.3. MYH7 R453C piglets developed malignant fibrosis

Myocardial fibrosis usually occurs in the end-stage of cardiovascular disease and greatly increases the risk of HF and SCD [22,23]. The left cardiac ventricular presented apparent fibrosis within the newborn MYH7 R453C piglets with the total fibrotic field of 37.77% in vision (figure 2*a,b*). Here, a significant decrease in microvessel density within the MYH7 R453C piglets was compared with WT piglets (figure 2*c,d*). The mRNA expression of profibrotic gene, Col1a1, Col3a1, TGF-β1 and CTGF were in line with the observation in the total histological section as there were elevated gene expression in MYH7 R453C compared with WT piglets (figure 2*e*). Western blotting was used to further evaluate the MYH7 R453C piglets on the expression of Bax and Bcl-2 in myocardial tissues. As shown in figure 2*f,g*, compared with the WT group, the expression of Bax in the MYH7 R453C group was significantly upregulated, and the expression of Bcl-2 was significantly downregulated. Compared with the WT group, the ratio of Bcl2/Bax is significantly decreased in the MYH7 R453C group. The upregulated expression of extracellular matrix proteins and pro-fibrotic genes future indicated the premature myocardial fibrotic remodelling in piglets with the MYH7 R453C mutation.

### 3.4. Preliminary mechanism exploration of transgenic MYH7 R453C piglets

**Figure 7.**
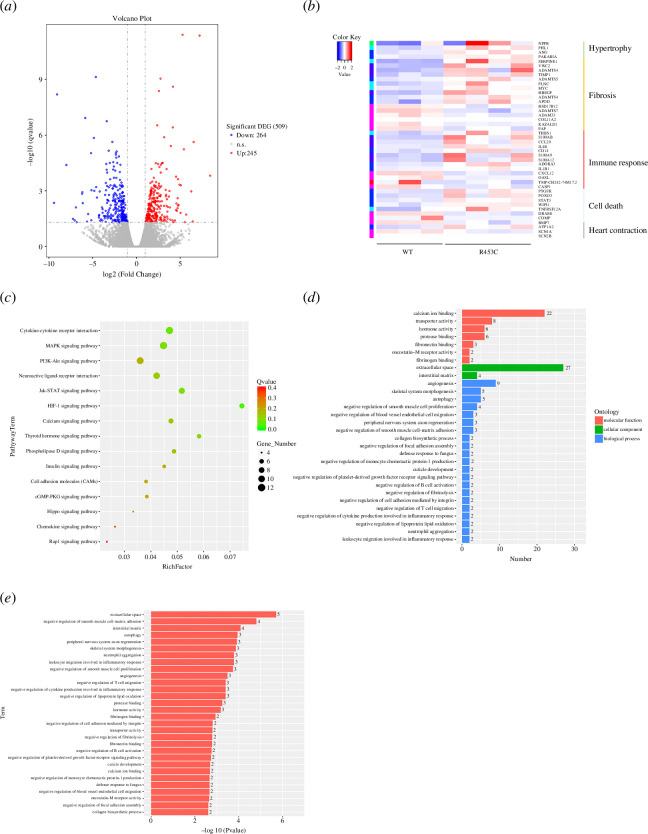
Transcriptome analysis of the transgenic MYH7 R453C porcine. WT (*n* = 3) and MYH7 R453C (*n* = 4). (*a*) The volcano plot of significant DEGs in MYH7 R453C piglets versus WT piglets. (*b*) Heat map of gene cluster involved in hypertrophy, fibrosis, inflammation and apoptosis. (*c*) The top 15 KEGG pathways of the most DEG enriched based on environmental information processing. (*d*,*e*) GO terms of DEGs within MYH7 R453C versus WT piglets upon bar plot (*d*) and *p* value (*e*).

To explore the inherent pathogenic mechanism of HCM caused by MYH7 R453C, RNA-seq was performed. With WT piglets as control, the number of differentially expressed genes (DEGs) within MYH7 R453C was 509 (figure 7*a*). Heat maps displayed that DEGs mostly enrich in cardiac hypertrophy, fibrosis, inflammatory response and apoptosis (figure 7*b*). The most enriched pathway terms of the MYH7 R453C piglets were the cytokine–cytokine receptor interaction, the PI3K-Akt and MAPK signalling pathway (figure 7*c*). Gene ontology (GO) analysis mostly enriched on the extracellular matrix, inflammation and the procedure of cell growth and apoptotic in MYH7 R453C transgenic piglets (figure 7*d,e*). In addition, western blotting analyses the expression of pivotal proteins, such as Akt, mTOR (for the PI3K-Akt pathway) and Ras, ERK1/2 (for the MAPK pathway) and key markers of apoptosis including activated-caspase 3, PARP and cytochrome *c*. The western blotting results show that these pathways are activated in the hearts of MYH7 R453C mutant piglets compared with the WT group (electronic supplementary material, figure S5). Taken together, we suspected that MYH7 R453C mutation influenced the PI3K-Akt, MAPK and apoptotic signalling pathways.

### 3.5. Effect of MYH7 R453C on fetal gene program *in vitro*

To more clearly delineate the pathological effects of MYH7 R453C mutation on cardiomyocytes, we constructed transgenic H9C2 cell lines with MYH7 R453C heterozygous mutation. The growth rate of gene-edited cells was lower than that of WT cardiomyocytes (figure 3*a*). Furthermore, no off-target mutations were detected at the five potential off-target sites in the MYH6/MYH7 R453C cell lines (electronic supplementary material, figure S6 and table S5). We then quantified the protein expression of MYH7 and found that there was a significant increase of β-MHC but not alpha-MHC within gene-edited rat cardiomyocytes (figure 3*b–d*). Similar to what was observed at the tissue level, mRNA expression of hypertrophic genes, NPPA, NPPB and MYH7 were significantly increased in the transgenic cell line (figure 3*e,f*). Meanwhile, the expression of profibrotic genes Col1a1, Col3a1, CTGF and TGF-β1 were all elevated in H9C2 with MYH7 R453C mutation (figure 3*j*). The results showed that MYH7 R453C promoted cardiac remodelling *in vitro*.

### 3.6. Effect of MYH7 R453C on oxidative stress *in vitro*

Cardiac remodelling is usually accompanied by oxidative stress. *In vitro*, we observed elevated production of ROS in H9C2 cell lines with MYH7 R453C mutation (figure 4*a*). Compared to WT H9C2, the expression of MDA was increased (figure 4*c*) while the levels of SOD and GSH were significantly decreased in MYH7 R453C mutants (figure 4*b,d*). The isoforms Nox2 and Nox4 of NADPH oxidase are found together in numerous cell types and play a role in redox-sensitive signal transduction triggered by agonists. Nox4 overexpression can substantially increase basal ROS generation. To explore the source of ROS, we examined the protein expression of Nox2 and Nox4 (figure 4*e,f*), the two major producers of ROS sources in both cardiac tissue and H9C2 cardiomyocytes. Though protein expression of Nox2 has no significant increase, the Nox4 was significantly upregulated in piglets and H9C2 cell line with MYH7 R453C compared to the WT (figure 4*g*). Above all, we proved that oxidation stress occurred within the MYH7 R453C cardiomyocytes and is mediated mainly by Nox4.

### 3.7. Verification of pathogenic mechanism *in vitro*

*In vitro*, we evaluated the activation of fibrosis, hypertrophy and inflammation-associated signalling pathways in the H9C2 cell lines. TGF-β is recognized as a fibrotic regulator and Smad2/3 is a pivotal downstream effector; the upregulated phosphorylation level of Smad2/3 in H9C2 with MYH7 R453C mutation demonstrated the activation of canonical TGF-β/Smad2/3 (figure 5*a–c*). The ERK1/2 protein was phosphorylated in the gene-edited cardiomyocytes (figure 5*a,d*), which was consistent with the individual. The increased phosphorylation of NF-kB p65 in gene-edited cardiomyocytes reflected the occurrence of inflammation response to mutation of MYH7 in cardiomyocytes (figure 5*e,f*). Taken together, these results suggest that the MYH7 R453C triggers the TGF-β and NF-κB-mediated signalling cascade in fibroblasts to drive their activation.

### 3.8. Epigallocatechin gallate reduces ROS production and cell death in MYH7 R453C mutation

**Figure 8.**
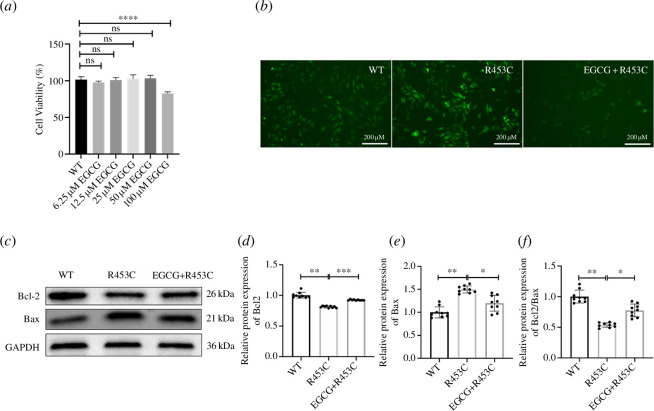
Effects of EGCG on the MYH7 R453C mutation H9C2 cells. (*a*) Effects of EGCG on the MYH7 R453C mutation H9C2 cell survival rate. (*b*) Protective effects of EGCG in MYH7 R453C mutation on ROS production. (*c*) Effect of EGCG on Bax and Bcl-2 activities of the MYH7 R453C mutation H9C2 cells. (*d*) Bcl-2 expression were quantified by densitometry and normalized to GAPDH levels. (*e*) Bax expression was quantified by densitometry and normalized to GAPDH levels. (*f*) Quantitative analysis of the ratio of Bcl-2 to Bax in protein expression was evaluated. Data are expressed as the mean ± s.d. *p < 0.05, **p < 0.01, ***p < 0.001, ****p < 0.0001. *n* = 3 biologically independent samples.

Epigallocatechin gallate (EGCG) is a unique plant compound thought to reduce inflammation, aid weight loss and help prevent heart and brain disease [24]. Therefore, we explored to treat MYH7 R453C mutation H9C2 cell line as a cardiac damage model with EGCG. Firstly, the toxicity of EGCG on H9C2 cells was evaluated by 3-(4,5-dimethylthiazol-2-yl)-2,5-diphenyltetra-zolium bromide (MTT). Incubation with 0–50 µm EGCG for 24 h did not exhibit any significant effects on the viability and proliferation of the cells. However, further treatment of H9C2 cells with 100 µm EGCG for 24 h can induce H9C2 cardiomyocyte damage (figure 8*a*). Thus, further analysis used solutions with concentrations of 50 µm EGCG. Then, treatment with EGCG significantly inhibited the upregulation of ROS fluorescence intensity in MYH7 R453C mutation H9C2 cells (figure 8*b*). To further determine the inhibitory effects of EGCG on cell apoptosis, western blotting was performed to examine the Bcl-2/Bax changes in H9C2 cells. Compared with WT H9C2, the expression of Bcl-2 was significantly increased and the expression of Bax was significantly decreased (figure 8*c–f*). Taken together, these findings show that the effect of EGCG protects MYH7 R453C mutation-induced apoptosis by the regulation of Bax and Bcl-2 expression in the apoptosis pathway.

## 4. Discussion

Patients with MYH7 R453C mutation typically develop premature clinical symptoms and severe HCM phenotype, such as cardiac remodelling and arrhythmia, significantly increasing the risk of heart failure and sudden death [6]. However, there is still no effective treatment, and the widely accepted approach is to find new therapeutic targets. Here, we generated MYH6 R453C and MYH7 R453C swine models and cardiomyocyte models by introducing Anc-BE4 max and one gRNA, simultaneously targeting the MYH6 and MYH7 locus within PFFs and H9C2. In this study, we found only MYH7 R453C transgenic swine models and H9C2 models developed remodelling, which was associated with the activation of TGF-β/Smad2/3 cascades, MAPK-ERK1/2 and NF-κB signalling pathway. In addition, excess accumulation of ROS in transgenic H9C2 cells was generated by Nox4, and they also accelerated the production of inflammatory responses and cardiac remodelling.

Previous studies have shown that the transition of Arg453 to Cys453 in β-MHC is a highly pathogenic mutation and associated with a high incidence of sudden cardiac death [25]. The R453C mutant may alter the structure of the myosin protein, affecting its interaction with actin and impairing the process of muscle contraction [5]. Additionally, the mutation may interfere with ATP binding and hydrolysis, essential processes for energy generation and muscle movement [5,8]. Here, we generated piglets with two genotypes: MYH6 R453C mutation and MYH7 R543C mutation. The MYH7 R453C mutant not only developed cardiac hypertrophy remodelling, but also severe pathological fibrosis and loss of cardiomyocytes, while the MYH6 R453C mutant showed milder HCM characteristics. Although there is no exact proportion of people with hypertrophic cardiomyopathy, patients with MYH7 R453C mutation have been reported as an inherited familial HCM worldwide while HCM patients carrying the MYH6 R453C variant were rare [25–28]. MYH6 encodes the α-MHC, which shares over 90% sequence homology with β-MHC but differs in its expression and functional roles within the heart. Although MYH6 mutations, including R453C, can potentially alter α-MHC function, the clinical impact appears to be less severe compared with MYH7 mutations. This discrepancy may be attributed to differences in the expression patterns of these myosin isoforms, with α-MHC being predominant in atrial myocardium and β-MHC in ventricular myocardium, and the intrinsic differences in their motor functions and energy efficiencies, highlighting the importance of isoform-specific effects on cardiac pathology [29].

Cardiac fibrosis is an ordinary pathological change in end-stage cardiovascular disease, which eventually leads to SCD and sudden HF [12]. In addition to the obvious myocardial fibrosis observed by histopathology, the mRNA expression of profibrotic TGF-β, CTGF, Col1a1 and Col3a1 were elevated in piglets and cardiomyocytes with MYH7 R453C mutation. TGF-β is extensively implicated in the pathogenesis of fibrosis, and it is rapidly upregulated and released to trigger TGF-β cascades when damage occurs. TGF‐β could transform cardiac fibroblasts to activated myofibroblasts in a Smad2/3-dependent way [13,30]. *In vitro*, we found H9C2 with the MYH7 R453C mutation showed significantly upregulated phosphorylation of TGF‐β/Smad2/3 compared with WT.

*In vitro*, ERK1/2 and NF-κB signalling pathways were both activated in transgenic MYH6 R453C and MYH7 R453C groups, the same as the situation *in vivo*. The ERK1/2 signalling pathway is one of the MAPK pathways, which are often activated in response to extracellular stresses, and inhibitors targeting the MAPK pathways may provide new therapeutic approaches to prevent fibrosis [22]. By inhibiting the ROS-dependent ERK1/2 signalling pathway, tetrandrine can enhance cardiac function and inhibit the process of hypertrophy development [23]. NF-κB pathway mediated inflammatory response in response to internal and external stimulation, and overexpressed ROS also mediated the phosphorylation of NF-kB p65. MD2 could directly bind to Ang II and mediate the development of cardiac inflammation and remodelling through the TLR4/NF-κB pathway [31]. Crocin treatment is reported to improve heart function and ameliorate fibrosis in the myocardium by inhibition of NLRP3-mediated pyroptosis via NF-κB pathway [32].

ROS is a by-product of normal cell aerobic metabolism and it plays a critical role in balancing intracellular signal transduction and internal environment stability of the body [33]. However, excessive ROS will induce cell apoptosis and even necrosis through oxidative stress response [34]. Previous reports have shown that Ang II could induce cardiac dysfunction by tipping the balance of the oxidative status of the heart via increasing the levels of ROS and inhibiting the survival mechanism of cardiomyocytes, which leads to the production of ROS and TGF, apoptosis of cardiomyocytes and cardiac fibrosis [35,36]. In MYH7 R453C cardiomyocytes, we detected overexpressed ROS produced by Nox4, and it induced the phosphorylation of NF-kB p65 and initiated the inflammation response to copy with cardiac remodelling. Nox4 has been implicated in the progress of cardiac remodelling, upregulation of Nox4 could promote cardiac remodelling via activating the downstream signalling pathways. In cardiac fibroblasts, protocatechuic acid alleviates Ang II-induced fibrosis by repressing the NOX4/ROS/p38 signalling pathway [36]. NADPH oxidase enzymes are important regulators of the heart’s physiological function and promising therapeutic targets for drug development by inhibiting the Nox4/ROS/NF-kB signalling pathway. EGCG is a powerful polyphenolic, chemopreventive compound isolated from green tea. It is well known for its antioxidant properties. In this study, we demonstrated that MYH7 R453C mutation enhanced ROS production in H9C2 cells, which could be reversed by EGCG. Meanwhile, the *in vitro* results indicated that EGCG can regulate the disordered expression of Bcl-2 and Bax in the MYH7 R453C mutation H9C2 cells.

**Figure 9.**
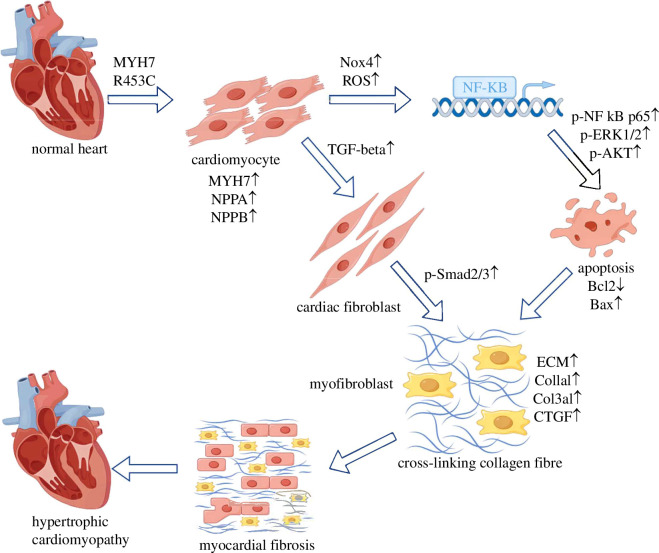
MYH7 R453C mutation upregulated TGF-β/Smad2/3, ERK1/2 and Nox4/ROS/NF-κB signalling cascade in cardiac fibroblasts, contributing to HCM myocardial fibrosis.

In conclusion, we generated swine and cardiomyocyte models with MYH6 R453C and MYH7 R453C mutation, and found MYH7 R453C (but not MYH6) mutation induced cardiac remodelling, inflammation and oxidation stress by the activation of the TGF-β/Smad2/3, ERK1/2 and Nox4/ROS/NF-κB signalling pathways (figure 9). We also showed that EGCG has a significant effect on reducing apoptosis by the regulation of Bax and Bcl-2 expression in MYH7 R453C mutation H9C2 cells, which provides valuable theoretical support for the study of therapeutic drugs of cardiac remodelling.

## 5. Conclusion

Recently, therapeutic targeting of fibrosis has been a hot topic. One effective solution is to target the inherent pathways. Here, we constructed two swine HCM models with the exhibition of pathological cardiac remodelling, breaking up the limitation of heterogeneous myosin isoform in mouse models. RNA-seq and western blotting for transgenic MYH7 R453C piglets and cardiomyocytes model proved that the malignant cardiac remodelling was associated with the activation of TGF-β/Smad2/3, ERK1/2 and NF-κB pathways. *In vitro*, we found that Nox4 contributed to excess ROS, and it stimulated inflammation response by activating the NF-κB signalling pathway. In addition, EGCG has a significant effect on reducing apoptosis by the regulation of Bax and Bcl-2 expression in MYH7 R453C mutation *in vitro*. Above all, these results provide a basis for us to study HCM drugs through alleviating cardiac remodelling, oxidation stress and inflammation via inhibiting canonical TGF-β/Smad2/3 cascades, non-canonical ERK1/2 signalling pathways and the Nox4/ROS/NF-κB signalling pathways.

## Data Availability

All data generated or analysed during this study are included in [36]. Supplementary material is available online [37].
